# Lung cancer-initiating cells: a novel target for cancer therapy

**DOI:** 10.1007/s11523-012-0247-4

**Published:** 2013-01-15

**Authors:** Brian J. Morrison, John C. Morris, Jason C. Steel

**Affiliations:** 1 Division of Hematology-Oncology, Department of Medicine, University of Cincinnati, Cincinnati, OH 45267 USA; 2 Division of Hematology-Oncology, Vontz Center for Molecular Studies, Department of Internal Medicine, University of Cincinnati, 3125 Eden Ave., M/L 0508, Cincinnati, OH 45267 USA

**Keywords:** Lung, Cancer, Cancer-initiating cells, Stem cells, Self-renewal, Targeted therapy, Immunotherapy

## Abstract

Lung cancer is a major public health problem causing more deaths than any other cancer. A better understanding of the biology of this disease and improvements in treatment are greatly needed. Increasing evidence supports the concept that a rare and specialized population of cancer cells, so-called cancer-initiating cells with stem cell-like characteristics, is responsible for tumor growth, maintenance, and recurrence. Cancer-initiating cells also exhibit characteristics that render them resistant to both radiation and chemotherapy, and therefore they are believed to play a role in treatment failure. This has led to the hypothesis that traditional therapies that indiscriminately kill tumor cells will not be as effective as therapies that selectively target cancer-initiating cells. Investigating putative cancer-initiating cells in lung cancer will greatly benefit the understanding of the origins of this disease and may lead to novel approaches to therapy by suggesting markers for use in either further isolating this population for study or for selectively targeting these cells. This review will discuss (1) lung cancer, (2) stem cells, and the role of cancer-initiating cells in tumorigenesis; (3) markers and functional characteristics associated with lung cancer-initiating cells; and (4) the potential to selectively target this subpopulation of tumor cells.

## Lung cancer and cancer-initiating cells

Lung cancer is the leading cause of cancer-related deaths worldwide [1]. Despite recent advances in treatment, the overall survival of patients with lung cancer remains only 15 % at 5 years, and this declines to less than 2 % in patients with metastatic disease [2]. For patients with advanced small cell lung cancer (SCLC), disease survival is 1–2 % [3]. Patients who respond to initial treatment often relapse and succumb to chemotherapy-resistant tumors [4]. This familiar clinical scenario is not restricted to lung cancer, but occurs in several hematological and solid tumors. Hence, new treatments that specifically target chemotherapy-resistant cells are needed.

The majority of solid tumors are composed of a heterogeneous population of cells including a proportion of cells that are innately chemotherapy-resistant or have gained resistance through acquired mutations (reviewed in [5]). As a result, chemotherapy predominantly kills the drug-sensitive cells, leaving behind a heterogeneous population of resistant cells, including those that have the potential to re-populate the tumor or seed new metastatic sites. These cells are putative cancer-initiating cells (CICs). We believe that under the selective pressure of chemotherapy the CICs, which are innately chemo-resistant, are able to produce progenitor cells which are also resistant to chemotherapy leading to the development of a treatment resistant tumor. Evidence for this can be seen in chemotherapy resistant tumors which have an acquired increased expression of the multi-drug resistance genes (e.g., MDR1), which encode for drug efflux pumps. These pumps are highly expressed in CICs and are thought to be a major factor in their innate drug resistance [6].

The concept of a specialized population of cells within tumors termed CICs, or alternatively cancer stem cells or tumor-initiating cells, has received considerable recent interest. CICs represent a subpopulation of transformed cells, distinct from more “differentiated” tumor cells, and are thought to be responsible for tumor organization, maintenance and progression, and resistance to therapy. CICs display functional characteristics of stem cells such as the capacity for self-renewal and the ability to give rise to differentiated progeny responsible for tumor proliferation. Putative CICs have been identified in acute myeloid leukemia (AML) [7, 8], and solid tumors of the brain [9, 10] and breast [11], among others. More recently, lung CICs have been isolated from human cell lines and patient samples [12–19]. CICs are posited to share many of the properties of normal somatic stem cells.

## Stem cells and the role of CICs in tumorigenesis

Normal somatic stem cells have the following attributes: (1) they are capable of self-renewal and repair in adult tissues; (2) they have the capacity for differentiation and the ability to generate a large number of multi-lineage progeny; (3) they are normally quiescent, but can be activated and proliferate in response to various stimuli to maintain tissue homeostasis; and (4) they have flexibility in applying these characteristics [20]. Somatic stem cells reside at the top of a hierarchy of tissue cells. Downstream of the stem cells is the more differentiated and rapidly proliferating progenitor/transit-amplifying cell progeny. Progenitor cells are capable of differentiating into mature cells of various lineages. Differentiated cells are specialized for specific roles and make up the bulk of the tissue.

Under the appropriate conditions, stem cells may undergo either asymmetrical division, to give rise to one transit-amplifying cell and another stem cell, or symmetrical division, to give rise to two stem cells or two transit-amplifying cells [20]. Asymmetric division provides continuation of the stem cell compartment while also producing the starting material for production of differentiated cells. Symmetric division provides flexibility during homeostasis: producing two stem cells or two transit-amplifying cells may either increase or decrease the stem cell pool depending on the needs of the system [21]. It has been suggested that the stem cell environment or niche may alter the stem cell pool by influencing how the stem cell divides and the plasticity of these cells [22]. The lung has a hierarchical organization that is thought to be regulated through the presence and activity of lung stem cells [23].

CICs are cancerous cells with many similar attributes as somatic stem cells including (1) self-renewal capacity, (2) the ability to differentiate and produce multi-lineage progeny that are tumorigenic and non-tumorigenic, (3) the capacity to establish and maintain tumors, and (4) flexibility in the application of these processes. Both cell types are long-lived and exhibit telomerase activity and active anti-apoptotic pathways [24]. Somatic stem cells and CICs demonstrate resistance to toxins and chemotherapeutics, often as a result of expression of the ATP-binding cassette (ABC) membrane transporter proteins [15, 25], and are believed to be relatively resistant to radiation due to their low proliferative indexes. Stem cells and CICs are also motile, thus allowing migration and homing of somatic stem cells, and metastatic spread for CICs [26]. Additionally, both somatic stem cells and tumors/CICs have niches—surrounding environments where bi-directional interactions between somatic stem cells/CICs occur that help maintain them.

**Fig. 1:**
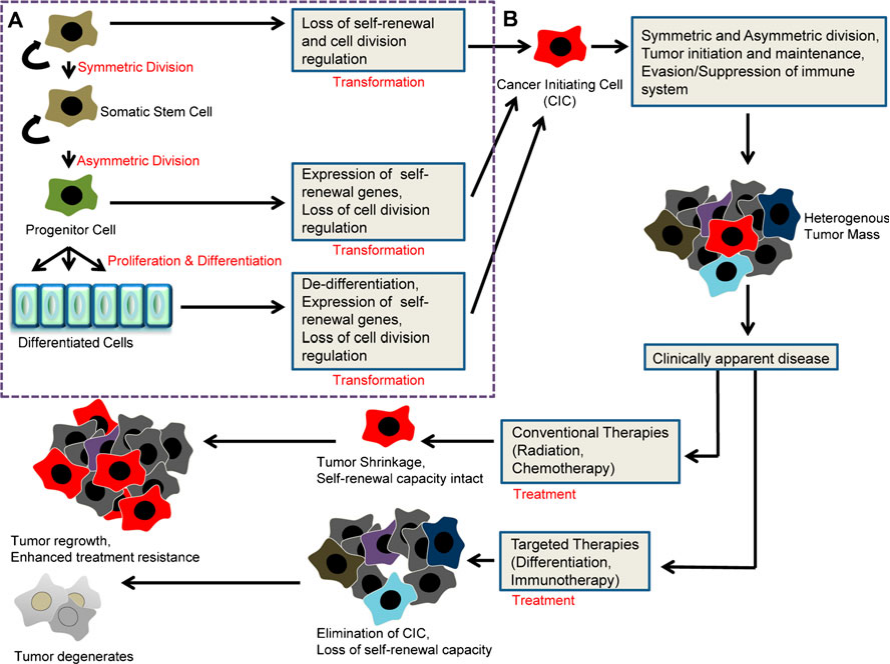
Origins of cancer-initiating cells and conventional therapy versus cancer-initiating cell targeted therapy. **a** CICs may be derived from somatic stem cells, progenitor cells, or differentiated cells. Somatic stem cells may require fewer transformational events than other cell types to become CICs. These events might include loss of regulation of self-renewal pathways and/or loss of cell division control. Somatic stem cells can undergo symmetric division to generate two stem cells, or asymmetric division to generate one stem cell and one progenitor cell. Progenitor cells may undergo a transforming event involving re-expression of self-renewal genes and a subsequent loss of cell division regulation to become CICs. Progenitor cells provide the starting material for proliferation and differentiation of cells into several lineages of differentiated cells. In order to generate a CIC, differentiated cells may undergo de-differentiation with re-expression of self-renewal genes and loss of regulation of cell division. **b** CICs form and maintain a heterogeneous tumor through rounds of asymmetric and symmetric division and through suppressing or evading host immune responses. Treatment typically begins soon after clinically apparent disease is detected. Conventional therapies that target differentiated cells, but spare CICs allow initial debulking of the tumor. However, tumor ultimately recurs because the rare therapy-resistant CICs have not been eliminated. These recurrences are often not responsive to subsequent treatment. Conversely, therapies that target CICs, but spare differentiated cells, do not appear to greatly debulk the tumor early on. However, as the CIC pool has been eliminated, the tumor can no longer maintain itself and ultimately degenerates

The origins of CICs are largely unknown (reviewed in [27]). It has been posited that normal stem cells that have undergone mutational events leading to a loss of self-renewal control are the source of CICs, or that transit-amplifying or differentiated cells that have undergone “de-differentiation,” acquired a self-renewal capacity, and lost regulation of cellular division could be the source of CICs (Fig. 1a). The similarity between somatic stem cells and CICs suggests that fewer steps might be involved for stem cells to transform into CICs compared to transit-amplifying or differentiated cells, which need to gain stem cell-like characteristics. However, the rarity of stem cells and the relative quiescent nature of somatic stem cells would suggest that they may be less likely to accumulate oncogenic mutations compared to transit-amplifying cell progeny. Regardless of the origin of CICs, they can be identified and isolated based on certain characteristics.

### Self-renewal pathways and stemness genes associated with CICs

One critical step in the transformation of a normal cell into a CIC is the acquisition of unregulated self-renewal. This is often associated with changes in the signaling pathways of Hedgehog (Hh), Notch, and Wnt/β-catenin, and/or the activation of “stemness” genes such as Oct-4, Nanog, Sox-2, or bone morphogenetic proteins (BMPs). Signaling pathways involved in maintaining CICs self-renewal or “stemness” may represent potential targets for cancer therapy.

The Hedgehog/Patched pathway is involved in embryonic growth and cell fate determination. During lung development, Hh signaling plays a role in lung bud branching morphogenesis [28]. Hh signaling has been implicated in regulating self-renewal of hematopoietic stem cells [29] and myeloid leukemia cells [30], as well as tumor cells in several solid tumors. Hh overexpression may lead to uncontrolled proliferation of tissue stem cells, generating a pool of target cells for additional oncogenic events leading to acquisition of CICs. Alterations in the Hh pathway have been reported in a variety of cancers including medulloblastoma, glioma, stomach, colon, pancreatic cancer, basal cell carcinoma, rhabdomyosarcoma, breast, prostate, and lung cancer [31].

The Notch family of transmembrane signaling proteins (Notch-1, -2, -3, and-4) regulate cell fate and are expressed in stem cells and transit-amplifying cells [32]. Notch signaling activation, via Jagged-1, is involved in the maintenance of self-renewal and plasticity for hematopoietic stem cells [33]. Inappropriate activation of Notch signaling stimulates proliferation, restricts differentiation and prevents apoptosis in cancer cells, and is associated with a variety of human cancers including breast, neuroblastoma, cervical, and lung [34]. Additionally, Notch signaling has been shown to preserve lung somatic stem cells in an undifferentiated state inhibiting terminal epithelial differentiation [35, 36].

The Wnt pathway is involved in cell fate determination in many organs during embryonic development. The Wnt pathway involves a large number of proteins in a cascade that ultimately leads to controlling the amount of β-catenin that reaches the nucleus to activate gene expression. Wnt signaling has been shown to regulate self-renewal in stem cells [37]. Evidence from transgenic mouse models demonstrated Wnt signaling pathway activation in stem cells leading to epithelial cancers [38]. Additionally, a pro-oncogenic role for β-catenin, a downstream target of Wnt signaling, has been described [39]. Drawn together, this indicates the involvement of Wnt signaling pathway members in the deregulation of stem cells into CICs. Evidence has emerged that Wnt signaling may play a role in lung tumorigenesis [40].

Induced stem cells are somatic cells that have been reprogrammed through forced expression of specific genes to obtain pluripotent stem cell-like characteristics [41]. This has led to the identification of “stemness” genes/transcriptional regulators involved in this process including c-myc, Klf4, Nanog, BMPs, Sox-2, and Oct-4. Sox-2 and Oct-4 in particular are involved in regulating self-renewal and pluripotent potential in stem cells and CICs. Enhanced expression of these genes, often in association with other functional/phenotypic markers or characteristics, has been used to characterize CICs from non-CICs in lung cancer [12–14, 17–19, 42].

## Biological markers and functional characteristics of lung CICs

Various biological markers have been used for isolating and characterizing lung CICs. These include (1) cell surface markers such as CD133 and CD44; (2) phenotypic characteristics such as exclusion of Hoechst 33342 dye (side population) and aldehyde dehydrogenase 1 (ALDH1) activity; and (3) functional characteristics such as chemo- or radiotherapy resistance, growth as spheroids in serum-free medium, and tumor initiation in vivo (see Table 1). These characteristics are often used in concert (in addition to demonstrating activation of self-renewal pathways and stemness genes) to isolate and identify putative lung CIC populations.

**Table 1:** Functional characteristics and markers for human lung cancer-initiating cells

	CIC-related characteristics	Citation
Functional characteristic		
Sphere growth in serum-free medium	Sphere growth from 7/19 patient lung tumor samples (SCLC and NSCLC) with varying frequency of CD133+ expression (0.6 % to 22.0 % of tumor cells that were capable of forming spheres). CD133+ lung cancer spheres demonstrated (1) expression of stemness genes such as OCT4 and NANOG, (2) self-renewal potential, (3) proliferation and differentiation ability (with subsequent loss of tumorigenic potential upon differentiation), (4) chemotherapy resistance, and (5) ability to recapitulate tumor heterogeneity in vivo	[14]
	Sphere growth from 10 NSCLC patient samples and five lung cancer cell lines sorted for CD133+ expression	[13]
	Sphere growth in 11 out of 15 lung adenocarcinoma malignant pleural effusion patient samples. Compared to adherent cells, sphere cells were associated with enhanced ALDH1 activity and Oct-4, Nanog, Notch3, and Stat3 mRNA expression	[19]
Chemoresistance	In the human lung cancer cell line H460, drug-selected cells (doxorubicin, cisplatin, or etoposide) demonstrated (1) spheroid formation; (2) self-renewal capacity and ability to differentiate; (3) expression of CD133; (4) enrichment for SP cells; (5) expression of embryonic stem cell markers, growth factor receptors, and chemokine receptors; and (6) high tumorigenic and metastatic potential	[18]
Cell surface marker		
CD133 (AC133, Prominin 1)	From patient lung cancer tissue samples, compared to CD133− cells, CD133+ cells displayed (1) enhanced expression of *OCT4*, (2) enhanced self-renewal ability, (3) increased expression of ABCG2, (4) enhanced resistance to chemotherapy and radiotherapy, (5) increased invasive ability, (6) increased in vivo tumor-restoration and proliferative capacity, and (7) increased spheroid formation	[13]
	From patient lung cancer tissue samples, compared to CD133− cells, CD133+ cells displayed (1) enhanced tumorigenic potential in vivo and (2) enhanced expression of ABCG2, CXCR4, α-6 integrin (CD49f), *OCT4*, and *NANOG*. CD133+ cells demonstrated a self-renewal capacity in vitro, and cisplatin resistance in vitro and in vivo	[12]
CD44	Expression of CD44 (62 % to 96 % of tumor cells) in 6/10 human NSCLC lines examined. Compared to CD44− cells, CD44+ cells displayed (1) spheroid formation, (2) resistance to cisplatin treatment in vitro, (3) enhanced tumorigenicity in vivo, and (4) enhanced expression of stemness genes *OCT4*, *NANOG*, and *SOX2*	[17]
Phenotypic marker		
ABCG2 activity (side population expression)	In human lung cancer cell lines (H460, H23, HTB-58, A549, H441, and H2170), compared to non-SP cells, SP cells demonstrated (1) enhanced invasiveness in vitro and tumorigenicity in vivo, (2) enhanced ABCG2 and human telomerase reverse transcriptase expression, and (3) resistance to multiple chemotherapy drugs	[15]
	In human SCLC cells (NCI-H82, H146, and H526), SP expression comprised <1 % of cells. Compared to non-SP cells, SP cells were associated with (1) higher proliferative capacity; (2) efficient self-renewal capacity; (3) decreased expression of differentiated cell markers; (4) enhanced tumorigenicity; and (5) expression of genes associated with CICs, including ABCG2, MYC, SOX1/2, WNT1, and Notch and Hedgehog pathway genes	[42]
Aldehyde dehydrogenase 1 (ALDH1) activity	In human lung cancer cell lines ALDH1 activity was associated with (1) capacity for proliferation; (2) self-renewal and differentiation; (3) resistance to chemotherapy; (4) expression of CD133; and (5) enhanced tumorigenicity, as well as ability to recapitulate the original tumor heterogeneity in vivo	[16]
	From 303 clinical patient specimens and controls, overexpression was positively correlated with stage and grade to tumor and associated with poorer prognosis for patients with early-stage lung cancer	

### CIC cell surface markers: CD133 and CD44

CD133 (prominin-1) is a five-transmembrane glycoprotein initially identified as a marker for isolating CD34+ human hematopoietic progenitor cells [43, 44]. CD133 has subsequently been used to identify and isolate somatic stem/progenitor cells of neural [45], epithelial [46], and endothelial origin [47], as well as their putative corresponding CICs [10, 14, 48–51]. The function of CD133 has not been well described and its ligand is currently unknown. It has been reported that CD133 may play a role in cell cycle regulation and proliferation of cells, but not necessarily tumor initiation [52]. CD133 has been used as a marker to examine lung CICs. In a study assessing CD133 in lung cancer cells from patients and cell lines, Chen et al. demonstrated higher expression of Oct-4 in CD133+ cells compared to CD133− cells [13]. In that study, Oct-4 expression was demonstrated to be essential for maintaining stem cell-like properties such as self-renewal capacity and invasiveness. Compared to CD133− cells, CD133+ cells were also reported to have enhanced resistance to conventional treatments and increased in vivo tumor-restoration capacity and proliferation. Eramo et al. demonstrated that CD133+ cells isolated from patient lung cancer samples can grow as tumorspheres and have a tumorigenic potential that is lost upon differentiation into CD133− cells [14]. In that study, the expression of CD133 ranged between 0.32 % and 22 % of tumor cells. CD133+ cells have been reported to have a low frequency in normal lung cells (<1 %) while a relatively high, but variable, frequency in most lung cancer samples; 47 out 56 primary lung cancer tissue samples demonstrated CD133 expression ranging from 0.02 % to 35 % [12]. Cells expressing CD133 have been described as having a self-renewal capacity, cisplatin resistance in vitro and in vivo, enhanced expression of stemness-related markers (Oct-4 and Nanog), and greater tumorigenicity [12]. However, CD133 is likely not a universal marker for all lung CICs. Leung et al. did not detect CD133 in nine out of 10 human non-small cell lung cancer (NSCLC) cell lines with an ability to induce tumors in immunocompromised mice [17]. Similarly, Meng et al. demonstrated that both CD133+ and CD133− subpopulations of two human lung cancer cell lines both contained CICs, calling into question the exclusive reliance on this marker [53].

CD44 is a cell membrane receptor glycoprotein that binds hyaluronic acid, is involved in cell adhesion, motility, and metastases, and, along with P-glycoprotein, has been linked to multi-drug resistance [54]. CD44 expression has been associated with up-regulation of other cancer-associated factors such as transforming growth factor-beta (TGF-β), genes associated with WNT signaling, cell adhesion molecules, and chemokines [55, 56]. CD44 can affect cell proliferation through its actions as a co-receptor with EGFR and ErbB family receptor tyrosine kinases [57]. Additionally, CD44 has been linked to enhancing anti-apoptosis through the PI3K/AKT cascade [58]. CD44 expression has been associated with human NSCLC cells enriched for CIC-like properties [17]. Using ten human NSCLC cancer cell lines, Leung et al. have shown that CD44+ cells have enhanced CIC properties compared to CD44− cells, including enhanced spheroid formation in vitro [17]. Enhanced in vivo tumor initiation compared to CD44− cells was shown for sorted CD44+ cells from the H1299, HKULC4, H1650, and HCC827 cell lines, with as few as 10,000 cells being capable of tumor initiation by days 30–68, compared to no tumors formed from the CD44− cells by day 90. For H1299 cells, CD44+ or unsorted cells were shown to have enhanced cisplatin resistance compared to CD44− cells. CD44+ cells, either freshly sorted or from CD44+-initiated tumors, showed expression of pluripotency/stemness genes (*OCT4*, *NANOG*, *SOX2*) not expressed by CD44− cells. However, CD44 expression was only detected in six of 10 cell lines examined [17], calling into question the exclusive reliance on this marker. The heterogeneity of CIC cell surface markers between tumors has led to the investigation of functional markers to identify CICs.

### Functional markers of lung CICs: side population and ALDH1 activity

A functional marker is a physical marker that can be used to target or isolate the CIC and is associated with a functional characteristic of CICs. One functional characteristic associated with CICs is resistance to cytotoxic agents. This resistance is attributed, in many cases, to increased expression of the ABCG2 multi-drug resistance transporter. ABCG2 is capable of pumping a variety of substrates, including cytotoxic drugs, out of the cell using ATP-dependent mechanisms [25]. Expression of these transporters is responsible, in part, for the common clinical problem of multi-drug resistance [59] and may protect CICs from cytotoxic agents used for cancer treatment. To model the function of ABCG2 transporters, the DNA-intercalating dye Hoechst 33342 has been used [25]. The efflux of this dye allows the identification of a population of cells, known as the side population (SP) by flow cytometric analysis. Goodell et al. first described the SP for hematopoietic stem cells [60] and the SP has subsequently been used to study the hierarchical organization of blood and other tissues. This functional property has also been used to study putative CICs from a variety of human cancers, including acute myelogenous leukemia as well as gliomas and neuroblastomas [61–63]. More recently, SP cells have been the focus of research examining lung CICs. Ho et al. demonstrated in a study of six human lung cancer cell lines that cells in the SP exhibited properties of CICs including (1) enhanced invasiveness and tumorigenicity, (2) increased expression of human telomerase reverse transcriptase and ABCG2 expression, and (3) resistance to chemotherapy [15]. Salcido et al. showed, in a study examining SP in three human SCLC cell lines, that compared to non-SP cells, SP cells demonstrated (1) a higher proliferative capacity, with efficient self-renewal capacity and decreased expression of differentiated markers; (2) increased tumorigenicity; and (3) increased expression of self-renewal genes such as *SOX1*/*2*, *WNT1*, *MYC*, and genes in the Notch and Hedgehog pathways [42].

Another functional marker of interest for isolating and characterizing CICs is aldehyde dehydrogenase-1 (ALDH1), a cellular detoxifying enzyme that oxidizes a variety of intracellular aldehydes to carboxylic acids [64]. The chemotherapeutics cisplatin and cyclophosphamide generate toxic aldehydes that are metabolized by ALDH1 [65]. High ALDH1 activity has been demonstrated in hematopoietic progenitor cells [66]. The activity of ALDH1 has also been detected in both normal and malignant human mammary stem cells and can be used to predict poor clinical outcomes in breast cancer [67]. Ginestier et al. demonstrated that high activity of ALDH1 identifies cells capable of self-renewal and high tumorigenicity in an immunocompromised mouse human tumor xenograft study [67]. In addition to hematopoietic and breast, activity of ALDH1 has subsequently been shown to enrich for normal stem cell populations or CICs in various other organ systems including brain, colon, and pancreas [68–70]. Recently, the activity and expression of ALDH1 has been correlated to the presence of lung CICs and the aggressiveness of lung tumors [16]. Jiang et al. has demonstrated using human NSCLC cell lines that cells expressing active ALDH1 are associated with a capacity for proliferation, self-renewal, and differentiation [16]. These cells also displayed expression of the CIC marker CD133, and CIC functional attributes of enhanced tumorigenicity and resistance to chemotherapy. In the same report, overexpression of ALDH1 was reported to correlate with poor prognosis for patients with early-stage NSCLC [16]. These findings make ALDH1 activity an attractive functional marker for identifying CICs, for use as a potential prognostic tool, and for use as a therapeutic target.

### Functional characteristics of lung CICs

CICs from various cancers have been shown to form multicellular three-dimensional spheres, also termed “tumorspheres” or “spheroids” when grown in vitro in non-adherent, serum-free media conditions [10, 14, 71]. In these conditions, most cells undergo a form of apoptosis known as anoikis induced by anchorage-dependent cells detaching from the surrounding extracellular matrix. However, rare anoikis-resistant cells divide and generate heterogeneous spheroid structures, composed of (1) cells that are differentiated and not anoikis-resistant, and (2) rare long-term proliferating anoikis-resistant cells—putative CICs. Sphere assays, and a recent mathematical interpretation of the sphere assay, allow for the assessment of the symmetric division expansion rate of malignant stem cell-like cells, and the evaluation of the effects of therapeutics on the self-renewal and proliferative activity of these cells [72]. Sphere assays are therefore powerful tools to assess the functional and phenotypic properties associated with putative CIC populations. Eramo et al., in a study of patient tumor samples, demonstrated sphere formation in seven out of 19 lung cancers [14]. In this study, long-term sphere culture was used to expand CICs for further analysis. Sphere-derived cells were found to have in vitro and in vivo properties of lung CICs including (1) self-renewal potential, (2) extensive proliferation and differentiation capacity, (3) expression of CD133, (4) expression of embryonic stemness genes such as *OCT4* and *NANOG*, and (5) chemotherapy resistance and tumorigenicity. Mancini et al. have demonstrated sphere growth in 11 out of 15 lung adenocarcinoma malignant pleural effusion patient samples [19]. Compared to matched adherent cells, sphere-derived cells demonstrated enhanced ALDH1 activity and expression of mRNA for Nanog, Notch3, Oct-4, and STAT3. Despite the lack of sphere-forming capability for all tumors evaluated in these studies, the in vitro sphere assay is useful for characterizing and isolating CICs. Spheroid culture and demonstration of long-term self-renewal as spheres is a routine trait characterized for putative lung CIC populations in a number of additional studies [12, 13, 17, 18].

Chemoresistance and radiation resistance are another functional characteristic associated with CICs. Chemoresistance often goes hand in hand with expression of functional markers such as SP expression, but warrants inclusion on its own merit. Chemotherapy resistance and expression of the SP/ABCG2 transporter has been used to enrich and characterize CICs. Levina et al. characterized drug-selected H460 human lung cancer cells and showed that these cells have characteristics of lung CICs including (1) sphere formation and self-renewal capacity; (2) an undifferentiated phenotype with an ability to differentiate; (3) expression of the SP, CD133, embryonic stem cell markers, and growth factor and chemokines receptors; and (4) a high tumorigenic and metastatic potential [18]. A variety of other studies have also used chemoresistance as a functional characteristic for CIC identification [12–17]. Less is known about radiation resistance and lung CICs. The existence of a subpopulation of radiation-resistant tumor cells has long been proposed by radiobiologists [73]. Characteristics of CICs that are thought to play a role in radiation resistance include, among others, (1) their relatively quiescent nature, (2) their capacity to regenerate tumors from a small starting number of cells, (3) more active DNA strand break repair pathways, and (4) down-regulation of senescence pathway associated with increased telomerase activity [74–77]. Addressing the mechanisms that cells use to become the treatment-resistant cell population may allow for specifically targeting these cells and increase the effectiveness of treatment.

Demonstration of CIC phenotypic markers and functional characteristics in vitro is often validated in vivo by tumor initiation studies. For human cancer cell lines/clinical samples, this is assessed through tumor formation from a limiting dilution of cells in immunocompromised mice. As all cells cannot induce tumors, the expansion of the resulting tumors is suggested to be driven by CICs. However, investigating CICs using xenograft mouse models for human tumor initiation is not without problems. Different strains of immunocompromised mice exhibit differing levels and types of residual immune effector cells. This in turn may alter the efficiency of tumor cell engraftment, and therefore the frequency or subpopulation of putative CICs may differ depending on the strain of immunocompromised mouse used. For instance, the detection frequency of tumorigenic cells in a melanoma xenograft model has been shown to be increased with the use of the NOD-SCIDγ (NSG, NOD.Cg-*Prkdc* ^*scid*^ *Il2rg* ^*tm1Wjl*^/SzJ) mouse compared to ordinary SCID mice that retain some natural killer cell activity, with single cell transplants capable of forming tumors in NSG mice [78]. Furthermore, the immunocompromised mouse microenvironment does not recapitulate the microenvironment in a human patient with naturally occurring cancer. While xenograft studies allow the identification of a sub-population of cells able to recapitulate a tumor in an immunocompromised mouse, they may not present an accurate picture of the characteristics of CICs. In order for a tumor to form in humans, potential CICs must interact with the immune system to prevent tumor recognition and elimination. This interaction is lost in immunocompromised xenografts. It has been suggested that studies investigating the ability of different subtypes of cells to grow in immunocompromised mice demonstrates selection for cells that can best adapt to growth in mouse tissue. Therefore, these studies might not be differentiating true tumorigenic CICs from non-CICs. Alternatively, immunocompetent syngeneic models allow for interactions of the recipient mouse host immune system, a situation that more closely models cancer in humans. Kelly et al. demonstrated that tumor growth was not necessarily driven exclusively by rare stem cell-like cells, when as few as 10 unsorted mouse lymphoma cells were reported to transplant disease in syngeneic recipient mice [79]. Recently, the tumorigenicity of lung CICs have been assessed in syngeneic mice to better model the interactions between the host immune system and CICs [80]. Syngeneic models allow the examination of CIC-derived cancer vaccines and immunotherapy, treatment that are unable to be assessed in immunocompromised animals. These caveats support the need for more rigorous testing of the cancer stem/initiating cell hypothesis, and the development of mouse models that either more closely recapitulates the human microenvironment for studying human cancer cells, or the use of syngeneic mouse models to test CICs from murine cancers.

## Targeting CICs

Resistance to chemotherapy and radiotherapy is thought to be behind the regeneration of tumor following initially successful treatment [81]. Compared to non-CICs, CICs are likely slow dividing, more resistant to apoptosis, and have an increased ability for DNA repair, making them more resistant to traditional methods of cancer treatment (reviewed in [74, 81]). This led to the hypothesis that therapies targeting differentiated cells, but sparing CICs, result in tumor relapse, while innovative treatments targeting CICs, but sparing differentiated cells, might lead to tumor eradication (Fig. 1b). Developing innovative treatments is an ambitious approach, as different CICs will likely respond with varying sensitivities to treatment. This is made more difficult by the lack of universal and specific markers for CICs. Pre-clinically, the sphere assay provides a model for assessing the treatment efficacy on the self-renewal and proliferative activity of CICs specifically that does not rely on identification of specific markers [72]. This could provide a model for the high-throughput assessment of various novel therapies targeting CICs. Treatments targeting CICs discussed here include (1) targeting pathways involved in self-renewal, (2) differentiation therapy, (3) antibody-directed and other targeted therapies for CICs, and (4) future directions for immunotherapy approaches to target CICs.

### Targeting CICs self-renewal pathways

There is increasing interest in investigating the self-renewal pathways utilized by CICs as therapeutic targets and combining these targeted CIC therapies with conventional treatments. These pathways include Hh, Notch, and Wnt.

The use of cyclopamine, a steroid-like molecule, to inhibit the Hh signaling pathway has shown promise in inhibiting the growth of medulloblastoma, basal cell carcinoma, and rhabdomyosarcoma [82, 83], and may find use in other tumors that are associated with aberrant Hh signaling. In SCLC, which has primitive features of pulmonary neuroendocrine cells, tumor development is dependent on activation of Hh signaling. Hh signaling plays a role in the normal differentiation of pulmonary neuroendocrine precursor cells and in the development of SCLC [84]. Hh pathway blockade by cyclopamine has demonstrated growth arrest and increased apoptosis for SCLC [84]. Therefore, some lung cancers may be susceptible to antagonists of the Hh pathway. A semi-synthetic derivative of cyclopamine, IPI-926, is currently undergoing clinical trials for various tumors [85].

Inhibition of Notch signaling can be accomplished at many different levels (reviewed in [86]) including, among others, the use of (1) receptor decoys that interfere with Notch/ligand interactions, (2) γ-secretase inhibitors to block Notch activation through proteolytic cleavage of the receptor by γ-secretase protein complex, and (3) antibodies that interfere with Notch signaling. In the case of lung cancer, inhibition of Notch signaling, in some NSCLC lines, has shown evidence of increasing apoptosis and decreasing tumor growth [87, 88], potentially through blocking the self-renewal efficiency of CICs. Haruki et al. demonstrated that a dominant-negative Notch-3 receptor, capable of inhibiting the Notch-3 pathway, was effective in reducing soft agar growth of human lung cancer lines and sensitized these lines to subsequent EGF receptor tyrosine kinase inhibition treatment [87]. Konishi et al. demonstrated that MRK-003, a gamma-secretase inhibitor, inhibited Notch-3 signaling and subsequently reduced growth and increased apoptosis of human lung cancer cell lines in vitro and in vivo using xenograft models [88]. Assessment of antibodies directed against Notch signaling is ongoing with results showing tumor inhibition and inhibition of cell fate decisions such as self-renewal in adult stem/progenitor cells [86].

The Wnt pathway can be inhibited through a variety of mechanisms. Targeting of β-catenin has received attention with retinoic acid (RA) [89] and tyrosine kinase inhibitors such as imatinib [90, 91] being shown to down-regulate β-catenin signaling. A monoclonal antibody directed against Wnt-1 has demonstrated an effect against a NSCLC cell line in vitro through inhibiting the Wnt/β-catenin signaling pathway and induction of apoptosis [92]. This monoclonal antibody also suppressed tumor growth in vivo for both non-established tumors and already established tumors.

The effect these treatments may have on somatic stem cells and other normal cells has to be weighed against their treatment efficacy against CICs. Targeted therapy against self-renewal pathway signaling molecules used by CICs may hold promise as a treatment, but to determine which patients are likely to benefit from treatment molecular tests assessing the signaling pathways activated in an individual tumor needs to be explored. This may be possible on a routine clinical basis as the cost of whole tumor genome expression analysis continues to fall. Designing rational treatment regimens targeting signaling pathways needs to be incorporated into current treatments to most effectively ablate self-renewal capacity in CICs and induce apoptosis of these cells leading to reduction in tumor burden.

### Targeting CICs using differentiation therapy

Another approach to target CICs is the induction of their differentiation, resulting in the loss of their ability for self-renewal and tumor maintenance. RA is one therapy used in the clinic to promote differentiation of epithelial cells [82]. RA-based therapy followed by chemotherapy has been used in acute promyelocytic leukemia and could also find use in solid tumor therapy [93]. The TGF-β superfamily member BMP-4 has been utilized to differentiate human glioblastoma stem cell-like CICs, with subsequent reduction in proliferation and induction of differentiation markers noted [94]. Recently, Azzi et al. demonstrated the use of interleukin (IL)-15 in the differentiation of renal CICs, resulting in the decrease of the CIC pool and generating differentiated non-tumorigenic cells that are susceptible to treatment with chemotherapy [95]. The use of IL-15 as a differentiation therapy is interesting in light of its other roles as an immune-regulating and anti-cancer cytokine [96]. Inducing differentiation in lung CICs may hold promise as a novel treatment approach.

### Targeting CICs using antibody-directed and other targeted therapies

**Fig. 2:**
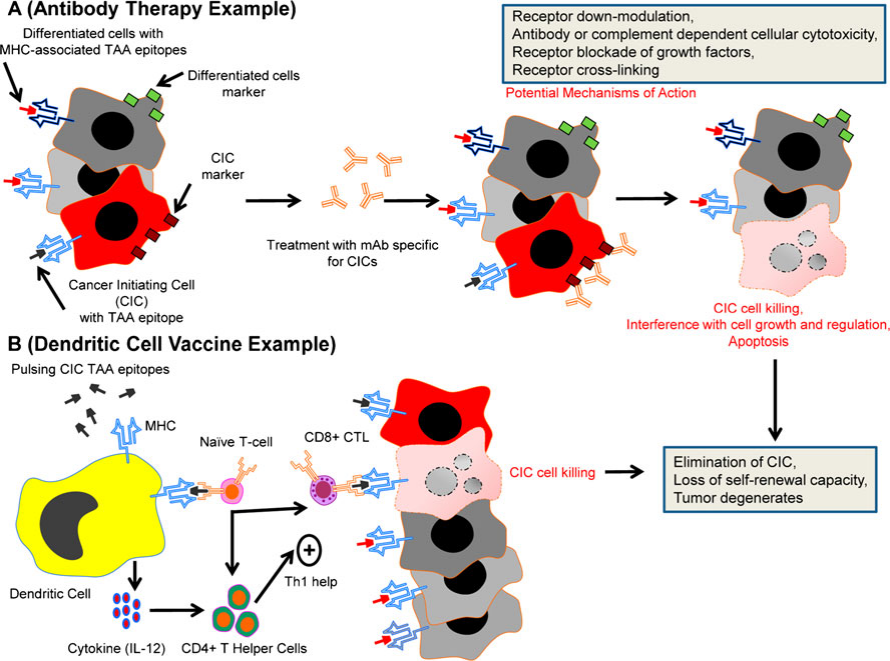
Targeted therapy for CICs. **a** CICs are likely to express specific markers (cell surface antigens in the example shown) that can be used for targeted therapy. One approach will be treatment with monoclonal antibodies (mAb) specific for markers expressed on CICs but not on differentiated cells. Through a variety of mechanisms, mAb therapy can lead to interference in cell growth or regulation of CICs or can induce apoptosis or cell killing directly. **b** Further characterization of lung CICs will likely reveal expressed antigens that can be used to target them. One cancer vaccine approach to eliminate CICs is shown here using antigen-presenting dendritic cells (DCs) pulsed with tumor-associated antigen (TAA) epitopes specific for CICs. DCs process and present TAA on major histocompatibility complexes (MHC) to T cells to stimulate a CD8+ cytotoxic T-lymphocyte (CTL) response that is thought to be critical for tumor elimination. DCs also express all of the necessary cytokine and co-stimulatory molecules, such as IL-12, to further direct the immune response to tumor and induce CD4+ Th1 T-helper cells. Once CICs are eliminated from the tumor the capacity for self-renewal is lost and the tumor degenerates. Note that tumor debulking of differentiated cells will also need to be accomplished concurrently

Antibody therapy directed specifically to CICs may find use in the clinic (Fig. 2a). The use of monoclonal antibodies (mAbs) targeting CICs is a relatively new approach. CICs are typically present in low frequency compared to non-CICs (typical estimates range between 0.1 % and 10 % of all tumor cells), and markers identifying CICs are not well characterized. Additionally, cell surface markers that have been characterized are not uniformly expressed by all CICs and often overlap with normal stem cells or non-tumorigenic cells. Identification of novel markers on CICs, preferably not expressed or with low expression on somatic stem cells or non-tumor cells, would be ideal. Additionally, therapy may be against the CIC itself (direct therapy) or against cells that maintain the CIC niche in the tumor (indirect therapy).

Direct mAb therapy specifically targeting lung CICs has not been well studied. However, two of the markers expressed by putative lung CICs, CD133 and CD44, do have the potential to be targeted through mAb therapy. AC133 is a mAb that recognizes CD133. In a pre-clinical study, AC133 conjugated to a cytotoxic drug was used to target hepatocellular cancer cells in vitro and in vivo with demonstration of tumor growth inhibition [97]. Similarly, an antibody directed against CD44, H90, has demonstrated efficacy in an AML model by specifically targeting CICs leading to promotion of differentiation, and inhibition of tumor proliferation and niche localization [98]. CD44 is a molecule with multiple variants, and future characterization of these variants is necessary for production of ideal mAbs for specific tumor types. Development of strategies to specifically target CD44/hyaluronan axis is ongoing (reviewed in [99]).

Stem cell niches are locations in a tissue that specifically support somatic stem cells and allow the repopulation of the stem cell compartment from stem cells or differentiated cells if the stem cell compartment is depleted [20]. Similarly, a tumor CIC niche specifically supports CICs. Therefore, it is possible that therapy that disrupts the tumor CIC niche could lead to the subsequent eradiation of CICs. Alternatively, tumor therapy that depletes CICs, but does not eradicate the tumor CIC niche, could lead to repopulation of the CIC pool.

Vascular endothelial cells are a component of the neural stem cell niche [100], and inhibitors of vascular endothelial growth factor (VEGF) have shown promise in ablating CICs, in addition to decreasing tumor vascularization, in a xenograft model of glioblastoma [101]. Anti-vasculature therapies include anti-VEGF antibodies (bevacizumab), anti-EGFR antibodies (cetuximab), and small molecules such as erlotinib that act on EGFR. CICs have been reported to express higher amounts of VEGF in both normoxic and hypoxic conditions compared to non-CICs. In a glioma model, high VEGF expression has been shown to lead to increased endothelial cell migration and tube formation, both of which can be subsequently blocked with bevacizumab [102].

Cells bearing the CXCR4 receptor molecule are capable of homing to specific niche environments via responding to a signal gradient of stromal cell-derived factor-1 (SDF-1/CXCL12). CXCR4 has been demonstrated to play a role in metastatic and drug-resistant lung CICs [103, 104] and is up-regulated in drug-selected lung CICs [18]. Thus, antagonists of the CXCR4/CXCL12 axis, such as Plerixafor (AMD3100) and T140 analogs (TN14003/BKT140), are of therapeutic interest for blocking metastatic disease, sensitizing tumor cells to chemotherapy, and targeting lung CICs [105].

Several mAbs have been utilized that neutralize autocrine signaling mediators involved in CIC growth and resistance to chemotherapy. In a preclinical model, an anti-IL-3 receptor alpha chain (CD123) neutralizing antibody demonstrated impairment of homing of leukemic CICs to the bone marrow, and a reduction in AML burden for mice with pre-established disease [106]. In a solid tumor model, an anti-IL-4 neutralizing antibody has been characterized for the ability to impair CICs in colorectal cancer [107, 108]. Todaro et al. demonstrated that CD133+ colon CICs utilize IL-4 to protect themselves from apoptosis, and that an anti-IL-4 neutralizing antibody will selectively sensitize these CICs to subsequent chemotherapy, thereby enhancing anti-tumor efficacy [107].

CICs are similar to normal somatic stem cells in many regards, meaning some treatments aimed at targeting CICs might put somatic stem cells at risk. ABC transporter (SP cells) inhibitors used in combination with chemotherapy have been shown to increase the efficacy of chemotherapy targeting CICs [81]. Also, an anti-ABCB5 antibody has been used to treat melanoma in a xenograft model resulting in reduction in tumor size and tumor eradication in 70 % of treated animals [109]. The proposed mechanism for this action was antibody-dependent cellular cytotoxicity (ADCC) directed against CICs. However, treatments targeting ABC transporters could also affect somatic stem cells, leading to potential toxicity in the bone marrow and improper maintenance of the blood–brain barrier [110].

Additional approaches exist to target CICs and these new strategies are the focus of ongoing research. Histone deacetylase inhibitors and other epigenetic-acting drugs are potentially useful for targeting CICs [111], as are small molecule inhibitors targeting key proteins in the intrinsic apoptotic pathway, a pathway often altered in CICs [112, 113]. Similarly, the knock-down of the “stemness” transcription factor Oct-4 has been shown to lead to apoptosis in CIC-like cells in a murine model of lung cancer [114].

### Targeting CICs using immunotherapy

Immunotherapy strategies utilize the patient’s own immune system in the treatment of cancer by inducing or enhancing (activation immunotherapies) immune responses to tumor or by suppressing (suppression immunotherapies) immune responses that are blocking effective tumor targeting. One promising activation immunotherapy strategy is the use of dendritic cell (DC) vaccines. DCs are powerful antigen-presenting cells that play a central role in initiating and directing immune responses through the processing of antigens and presentation of epitopes in the context of surface MHC molecules to T cells. DCs are equipped with all of the necessary co-stimulatory and cytokine signals to stimulate cytolytic T-lymphocyte (CTL) responses to tumor-associated antigens (TAA), leading to the elimination of tumors expressing these antigens (Fig. 2b). Cancer vaccines targeting solid tumors have been employed, with varying success, both pre-clinically and clinically in the treatment of cancer [115]. Several clinical trials have examined the efficacy of epitope-modified DCs, often accomplished by “pulsing” DCs ex vivo with synthetic TAA peptides [116, 117]. One drawback of peptide loading is the need for knowledge of the antigen sequence and the requirement for favorable binding affinity of the peptide for MHC. An alternative strategy is to load DCs with protein lysates generated from tumors. Tumor lysates, however, contain many antigens, most of which are not tumor specific.

Vaccine approaches that specifically target CIC antigens, and therefore CICs, may be more successful [118]. In a pre-clinical trial using irradiated CICs as antigens for a DC vaccine for glioblastoma, activation of antigen specific T cells and γ-interferon production was seen in the vaccinated animals [119]. These immune responses were correlated with prolonged survival in animals bearing tumor compared to an equivalent vaccine derived from non-CICs. In another pre-clinical study targeting murine CT26 colon cancer, Mori et al. compared vaccination against TAA expressed by CIC versus those expressed by non-CIC. Vaccination against non-CIC antigens did not induce an anti-tumor response whereas vaccination against antigens expressed on CIC led to a significant antitumor response [120]. These results highlight the importance of targeting antigens on CICs for an effective vaccine strategy.

## Concluding remarks

Investigating CICs are not without potential difficulties, including among others, (1) defining CICs and their markers, (2) the use xenotransplantation models, (3) identifying and measuring CIC stemness, and (4) the challenge of developing therapies that specifically target CICs (for a review of these see [121]). Studying lung CIC may have a major impact on cancer treatment by suggesting novel therapeutic approaches; however, additional studies are still required to further characterize the plasticity, heterogeneity, immune-modulating properties, and functional/physical phenotype of lung CICs. These studies will aid in identifying therapies that can specifically target lung CICs. Prospective clinical investigations assessing putative lung CIC markers and functional characteristics should be performed, with a focus on assessing cells with these characteristics before, during, and after treatment. In addition to suggesting new targets for therapy, these studies may provide diagnostic and prognostic information. Targeted therapy against CICs may need to be personalized for each patient, either through assessing the appropriate mAb or differentiation therapy required for their particular CIC phenotype, or through application of immunotherapy. To be effective, new therapies targeting CICs will need to be incorporated into clinical practice alongside traditional therapies that debulk the tumor and therapies that aim to interfere with the supportive niche of CICs, including anti-angiogenic or anti-stroma therapy. In this setting, the development of targeted CIC therapies could lead to meaningful increases in clinical responses.
